# MicroAIbiome: Decoding Cancer Types from Microbial Profiles Using Explainable Machine Learning

**DOI:** 10.3390/microorganisms13092210

**Published:** 2025-09-21

**Authors:** Md Motiur Rahman, Shiva Shokouhmand, Saeka Rahman, Nafisa Nawar Tamzi, Smriti Bhatt, Miad Faezipour

**Affiliations:** 1 School of Engineering Technology, Electrical and Computer Engineering Technology, Purdue University, West Lafayette, IN 47907, USA; rahma112@purdue.edu (M.M.R.); sshokouh@purdue.edu (S.S.);; 2 Department of Fishing & Post-Harvest Technology, Chattogram, Veterinary and Animal Sciences University, Chattogram 4225, Bangladesh; nafisa@cvasu.ac.bd; 3 Department of Computer & Information Technology, Purdue University, West Lafayette, IN 47907, USA

**Keywords:** microbiome, cancer classification, microbial signatures, machine learning, SHAP values

## Abstract

Microbial communities within human tissues are increasingly recognized as promising biomarkers for cancer detection. However, leveraging microbiome data for multiclass cancer classification remains challenging due to its compositional structure, high dimensionality, and lack of model interpretability. In this study, we address these challenges by introducing MicroAIbiome, a machine learning-based artificial intelligence (AI) pipeline designed to classify five cancer types such as esophageal carcinoma (ESCA), head and neck squamous cell carcinoma (HNSC), stomach adenocarcinoma (STAD), colon adenocarcinoma (COAD), and rectum adenocarcinoma (READ), using genus-level microbial relative abundances. Our pipeline incorporates zero-replacement, centered log-ratio (CLR) transformation, correlation filtering, and recursive feature elimination (RFE) to enable robust learning from compositional data. Among five evaluated classifiers, XGBoost achieved the highest accuracy of 78.23%, outperforming prior work. We further enhance interpretability using SHapley Additive exPlanations (SHAP)-based feature attribution to uncover class-specific microbial signatures, such as Corynebacterium in ESCA and Bacteroides in COAD. Our results highlight the importance of compositional preprocessing and explainable AI in advancing microbiome-based cancer diagnostics.

## 1. Introduction

Cancer remains one of the most urgent global health challenges, causing nearly 10 million deaths annually [1,2]. Despite advances in treatment and screening, early detection and accurate classification across diverse cancer types remains difficult [3,4,5]. Traditional approaches often overlook subtle, non-obvious biomarkers shaped by genetic, environmental, and immune interactions [6,7].

The human microbiome has recently emerged as a promising source of such biomarkers. Tumor-associated microbial communities can influence carcinogenesis through chronic inflammation, immune modulation, and metabolite production, and exhibit distinct signatures across cancer types and anatomical sites [8,9,10,11,12,13]. Importantly, microbiome profiles offer non-invasive accessibility and reflect early molecular changes in host tissues, supporting their potential as complementary diagnostic tools [10,14,15]. Resources like The Cancer Microbiome Atlas (TCMA) further enable systematic exploration of microbial patterns across multiple cancers [16,17].

Machine learning (ML) methods have been increasingly applied to microbiome-based cancer classification [18,19,20]. However, three major challenges persist: (i) the compositional nature of microbiome data complicates direct use of standard ML algorithms [21,22]; (ii) most prior studies are limited to binary classification, hindering generalization across anatomically related cancers [23,24]; and (iii) limited model interpretability reduces biological and clinical trustworthiness [25,26].

To address these issues, we introduce MicroAIbiome, a comprehensive machine learning-based artificial intelligence (AI) pipeline for multiclass cancer classification using genus-level microbial profiles. Our contributions are threefold: (i) we develop a compositional data-aware preprocessing pipeline that includes zero-replacement, centered log-ratio (CLR) transformation, correlation filtering, and recursive feature elimination (RFE); (ii) we benchmark five common classifiers, such as Support Vector Machine (SVM), eXtreme Gradient Boosting (XGBoost), Random Forest (RF), Logistic Regression (LR), and K-nearest Neighbors (KNN), within a rigorous multiclass framework; and (iii) we employ SHAP (SHapley Additive exPlanations)-based interpretation to identify key microbial genera associated with each cancer type. This work demonstrates the potential of microbiome-based cancer diagnostics using explainable AI and provides a methodological foundation for future efforts in non-invasive cancer detection and the discovery of microbial biomarkers.

## 2. Related Works

The intersection of microbiome research and cancer detection has gained momentum with the advent of next-generation sequencing (NGS), enabling sensitive and large-scale profiling of microbial DNA in tumor samples. However, this increased sensitivity also heightens the risk of contamination, particularly in low-biomass tissues, necessitating stringent quality controls and computational strategies to extract biologically meaningful patterns [18].

Machine learning (ML) and deep learning (DL) frameworks have shown considerable promise in modeling the complex relationships between microbial signatures and disease states. Classical algorithms such as Support Vector Machines (SVMs), Random Forest (RF), and Logistic Regression (LR) have been widely employed in cancer classification tasks, due to their ability to handle high-dimensional data and provide baseline interpretability [18,27,28,29,30]. More recently, gradient boosting models (e.g., XGBoost, LightGBM) and deep neural networks (DNNs) have demonstrated strong predictive performance, particularly in colorectal and breast cancer detection tasks [31,32,33,34,35].

Despite these advances, three limitations frequently recur in the literature. First, most existing models are trained on binary tasks, such as cancer versus healthy or two specific cancer types, which oversimplifies the diagnostic space and limits their real-world applicability. Second, few studies address the compositional nature of microbiome data, which violates assumptions of standard learning algorithms and introduces spurious correlations, unless properly transformed. Third, model interpretability remains limited, with many approaches treating ML models as black boxes, thereby hindering the biological relevance and clinical trustworthiness of the results.

Some recent works have incorporated preprocessing pipelines that involve dimensionality reduction or filtering via mutual information, Principal Component Analysis (PCA), or Least Absolute Shrinkage and Selection Operator (LASSO) [36,37,38]. However, these often overlook compositional constraints or ignore feature redundancy, both of which are central to microbiome analysis. Additionally, the use of post hoc interpretability tools such as SHapley Additive exPlanations (SHAP) and Local Interpretable Model-Agnostic Explanations (LIME) has only recently been explored in this domain, with few studies quantifying the impact of individual microbial features on model predictions across classes [39,40,41].

In contrast, our study uniquely combines a robust compositional data preprocessing pipeline with an extensive comparative evaluation across five ML algorithms in a multiclass classification setting. By integrating recursive feature elimination (RFE) and SHAP analysis, we enhance predictive accuracy, improve interpretability, and identify cancer-specific microbial biomarkers, such as *Bacteroides*, *Corynebacterium*, and *Helicobacter*. Our approach builds on prior efforts but extends them through both methodological rigor and biological insight.

## 3. Methods

### 3.1. Dataset

The microbiome dataset used in this study was obtained from The Cancer Microbiome Atlas (TCMA) [13], an open-access resource that provides microbial abundance profiles associated with various cancer types. The dataset comprises genus-level relative abundance profiles derived from tumor tissue samples, totaling 620 samples across multiple cancer types. For each sample, microbial composition is quantified based on next-generation sequencing data, and users may select taxonomic resolution ranging from phylum to genus. In this study, we focused on genus-level profiles, capturing 221 distinct microbial genera.

TCMA organizes the data into two complementary components: (1) a microbial dataset containing per-sample genus-level relative abundance values, and (2) a metadata file that provides cancer type annotations, sample identifiers, and associated TCGA (The Cancer Genome Atlas) project codes. For our analysis, we considered five cancer types with sufficient representation: esophageal carcinoma (ESCA), head and neck squamous cell carcinoma (HNSC), stomach adenocarcinoma (STAD), colon adenocarcinoma (COAD), and rectum adenocarcinoma (READ).

We focused on genus-level profiles because species-level resolution in TCMA was sparse and inconsistent across cohorts. Aggregating data at this level provided a more stable taxonomic resolution for reliable multiclass modeling, while also reducing data sparsity and batch-related artifacts.

### 3.2. Data Preprocessing

**Figure 1:**
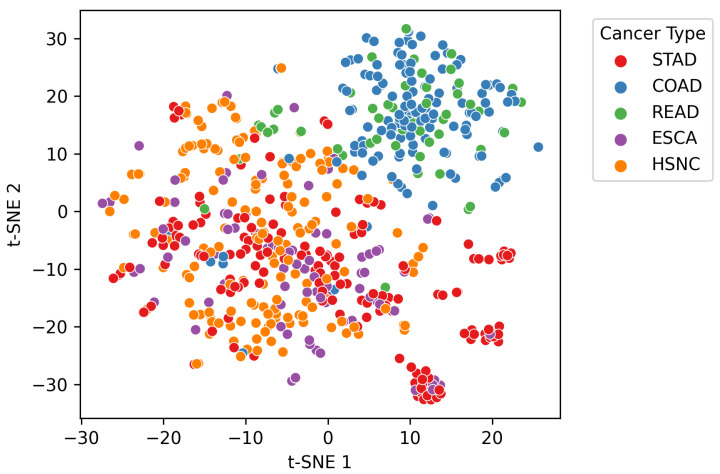
t-SNE (t-distributed Stochastic Neighbor Embedding) visualization of genus-level microbial profiles colored by cancer type. Each point represents a patient sample, and distinct clusters reflect differences in microbial composition across ESCA, HNSC, STAD, COAD, and READ.

**Figure 2:**
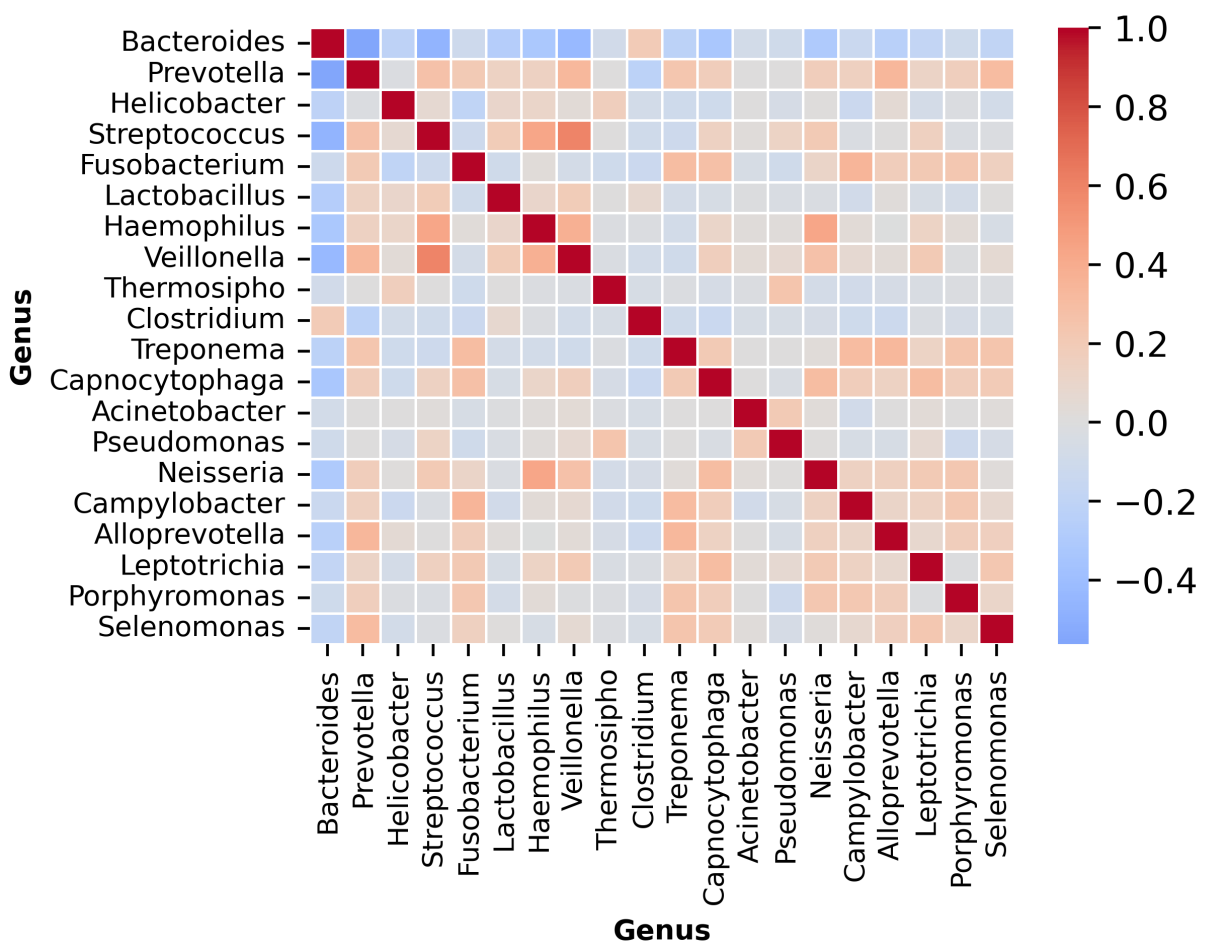
Microbial co-occurrence heatmap showing the top 20 genera across all cancer samples. Colors represent Spearman correlation coefficients, with red for positive and blue for negative associations. Positive correlations indicate co-occurring taxa, while negative ones highlight mutually exclusive genera. Notable patterns include the clustering of Bacteroides and Prevotella, which are dominant in gastrointestinal cancers.

**Figure 3:**
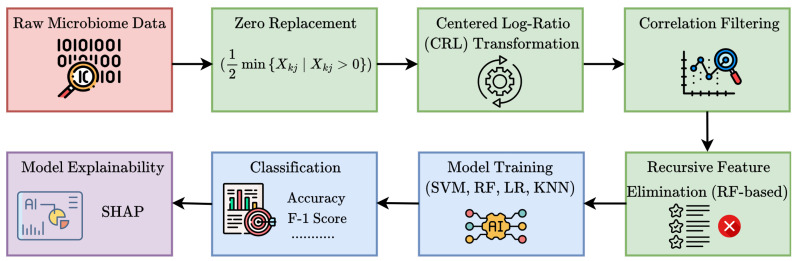
Workflow of the proposed MicroAIbiome pipeline for cancer detection.

The input dataset comprised genus-level relative abundance profiles obtained from microbial sequencing of tumor tissue samples across multiple patients. Each sample was linked to a specific cancer type, identified through manual mapping of anonymized patient identifiers. A t-distributed Stochastic Neighbor Embedding (t-SNE) visualization of the dataset is provided in Figure 1, illustrating distinct microbial composition patterns across cancer types. Additionally, the microbial co-occurrence heatmap of the top 20 genera (Figure 2) reveals strong inter-feature correlations, highlighting the need for careful preprocessing to mitigate multi-collinearity before model training. The complete workflow of the proposed *MicroAIbiome* pipeline, from preprocessing to classification and interpretation, is summarized in Figure 3.

Due to the compositional nature of microbiome data, a sequence of transformations was applied to enable the use of machine learning models. First, all zero entries in the abundance matrix were replaced using a minimal positive value strategy. Specifically, for each feature, zero values were imputed with half the smallest non-zero value observed in that feature, as shown in Equation (1). This approach maintains relative structure while ensuring numerical stability in downstream logarithmic operations.

(1) $$X_{ij}=\left\{\begin{array}{cc} \frac{1}{2}\min\left\{X_{kj}|X_{kj}>0\right\} & \mathrm{if}\;X_{ij}=0\\ X_{ij} & \mathrm{otherwise} \end{array}\right.$$

Here, $X_{ij}$ denotes the abundance of feature *j* for sample *i*. The expression $\min\left\{X_{kj}|X_{kj}>0\right\}$ identifies the smallest non-zero value for feature *j* across all samples *k*, where zero values are replaced with half of this value. If $X_{ij}$ is already non-zero, it remains unchanged. This step ensures that log-ratio transformations can be safely applied without encountering undefined values due to logarithms of zero. We applied this zero-replacement strategy because microbiome datasets are often sparse, with many zero entries arising from detection limits or low-abundance taxa. Direct application of log-ratio transformations on such data would result in infinite or undefined values. By replacing zeros with a small, feature-specific pseudo-count, we preserved the relative structure of the data while enabling stable and valid application of the centered log-ratio (CLR) transformation. This data-adaptive imputation minimizes the distortion of rare microbial features and ensures numerical robustness during downstream compositional analysis and model training.

To address the closure constraint inherent in compositional data, we applied the *centered log-ratio (CLR) transformation* to the abundance matrix $X\in R^{n\times d}$, where *n* is the number of samples and *d* is the number of microbial taxa. For a given sample vector $x_{i}=\left[x_{i1},x_{i2},\ldots ,x_{id}\right]$, the CLR-transformed vector $z_{i}$ is given by Equation (2):

(2) $$z_{i}=\left[\log\left(\frac{x_{i1}}{g_{i}}\right),\ldots ,\log\left(\frac{x_{id}}{g_{i}}\right)\right],\;\mathrm{where}\;g_{i}={\left(\prod_{j=1}^{d}x_{ij}\right)}^{1/d}$$

Here, $g_{i}$ is the geometric mean of the taxa abundances for sample *i*. By transforming each feature relative to the geometric mean, the CLR transformation maps the data from the simplex to real Euclidean space, thereby enabling the use of conventional statistical and machine learning models that assume unconstrained input.

After transformation, we performed correlation filtering to remove redundant features that exhibited high collinearity. Pairwise Pearson correlation coefficients were calculated, and for any pair of features with a correlation coefficient exceeding 0.7, one feature was removed. This step mitigates multi-collinearity, reduces dimensionality, and improves the interpretability and stability of downstream models.

Finally, the resulting feature matrix was split into training and test sets using an 80/20 stratified split, ensuring that all cancer types were proportionally represented in both subsets. Stratification preserves class balance, which is particularly important in multiclass classification tasks with imbalanced class distributions.

### 3.3. Contamination Handling

Because TCMA relies on the sequencing of low-biomass tumor samples, contamination remains a potential concern. To mitigate this, TCMA applies stringent filtering to distinguish tissue-resident microbiota from likely contaminants [13]. Within our pipeline, compositional transformations (e.g., CLR), correlation filtering, and recursive feature elimination (RFE) further reduce the influence of low-abundance and redundant taxa, thereby limiting spurious associations. While these steps enhance robustness, we acknowledge that residual contamination cannot be completely excluded. Future work should incorporate explicit contamination detection frameworks (e.g., decontam) and batch-aware preprocessing to further strengthen reliability.

### 3.4. Model Training and Evaluation

We implemented a comparative classification framework using five machine learning algorithms: RF, SVM, XGBoost, Logistic Regression, and KNN. Each classifier was embedded in a pipeline that included standardized preprocessing and feature selection procedures. Feature values were standardized to zero mean and unit variance using z-score normalization. Subsequently, recursive feature elimination (RFE) was applied to select a subset of informative features. RFE was driven by a Random Forest (RF) estimator and retained approximately 60% of the original features, which were chosen based on their predictive importance in a backward elimination process.

Model hyperparameters were optimized via *Grid Search* using *Stratified 5-Fold Cross-Validation*, which preserved class distributions across folds. The following parameter ranges were considered for each model: for Random Forest, number of trees and maximum depth; for SVM, regularization strength *C* and kernel coefficient γ; for XGBoost, tree depth and boosting rounds; for Logistic Regression, $L_{2}$ regularization strength; and for KNN, the number of neighbors.

## 4. Results and Discussion

### 4.1. Overall Performance

We performed a comprehensive comparison of five classifiers—Random Forest, SVM with Radial Basis Function (RBF) kernel, XGBoost, Logistic Regression, and KNN—using a meticulous preprocessing and feature selection process. The final models were retrained on the entire training set and evaluated on a separate test set. The overall results are summarized in Table 1.

Among all models, XGBoost achieved the highest test accuracy (78.23%) and macro-F1 score (0.75), followed closely by SVM with 77.42% accuracy and a macro-F1 of 0.70. Logistic Regression also performed competitively with 76.61% accuracy, highlighting the robustness of linear classifiers under CLR-transformed data. In contrast, Random Forest achieved the lowest test accuracy (70.97%), while KNN achieved 73.39% accuracy.

Most importantly, SVM achieved the highest Area Under the Receiver Operating Characteristic curve (AUROC, 0.95) and the lowest Brier score (0.0626). AUROC reflects the model’s ability to correctly rank true classes above false ones across all thresholds. Values closer to 1.0 indicating stronger class separation. A low Brier score shows that the predicted probabilities align well with actual outcomes, meaning if the model predicts a 70% chance of a cancer type, the event happens approximately 70% of the time. This reliable probability calibration is essential in clinical decision support, where thresholds for triage or risk stratification often depend on probability estimates rather than simple class labels. Meanwhile, XGBoost delivered the best balance of accuracy, F1, and Area Under the Precision–Recall Curve (AUPRC). AUPRC is important in imbalanced clinical datasets and emphasizes the model’s performance in correctly identifying positive cases. Therefore, while XGBoost excelled in overall classification performance, SVM offered the most trustworthy probability estimates, highlighting complementary strengths depending on whether the main goal is overall classification accuracy or clinically calibrated risk prediction.

### 4.2. Nested Cross-Validation Performance

To prevent data leakage during preprocessing steps (zero-replacement, CLR transformation, correlation filtering, and RFE), we used MicroAIbiome within a nested cross-validation (CV) framework. All preprocessing and feature selection procedures were limited to the training folds of the inner loop, while the outer loop provided unbiased estimates of generalization performance. Additionally, we conducted nested 5-fold cross-validation with hyperparameter optimization (Table 2) to support test set evaluation.

The nested CV results strongly confirmed the trends seen in the held-out test set: XGBoost and SVM consistently achieved the highest mean accuracies (76.9% and 75.8%, respectively) and macro-F1 scores, while Random Forest and KNN showed comparatively lower performance. The close match between nested CV estimates and test results demonstrates that our pipeline generalizes well and does not suffer from overfitting. This consistency further supports the reliability of the reported test performance and highlights the effectiveness of the MicroAIbiome preprocessing framework across multiple classifiers.

### 4.3. Class-Wise Performance

**Figure 4:**
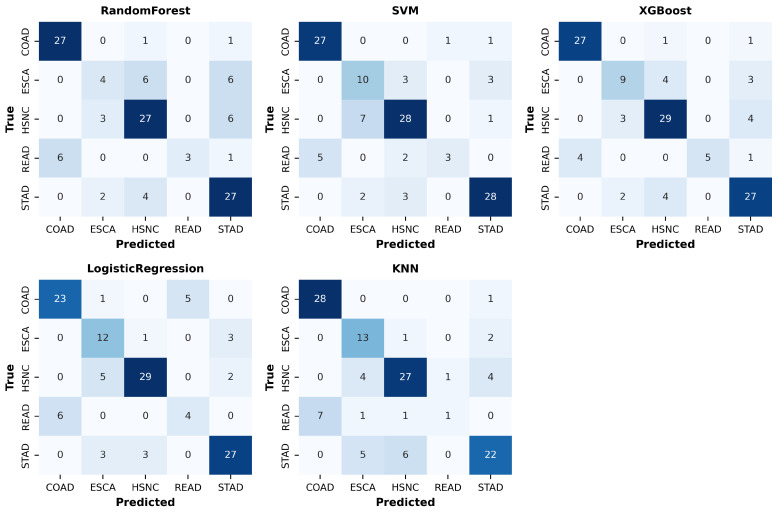
Confusion matrices for five classifiers (Random Forest, SVM, XGBoost, Logistic Regression, and KNN) on the test set. Diagonal cells indicate correct classifications, while off-diagonal cells show misclassifications. COAD and STAD were predicted more accurately, whereas ESCA and READ exhibited higher misclassification rates, reflecting class imbalance challenges.

Per-class results (Table 3) and confusion matrices (Figure 4) reveal systematic challenges in classifying minority classes, particularly READ and ESCA. Random Forest achieved high Recall for COAD (0.93) but very poor Recall for ESCA (0.25). KNN displayed unusually high Recall for ESCA (0.81) but failed almost completely to capture READ (Recall = 0.10). Logistic Regression and XGBoost offered more balanced performance, though they still struggled with minority classes (READ Recall = 0.40 and 0.50, respectively). SVM provided the most stable per-class trade-off, with Precision and Recall values above 0.75 for most classes, though Recall for READ remained low at 0.30.

These results highlight that while compositional-aware preprocessing (CLR transformation and correlation filtering) enhances overall performance, class imbalance remains a significant challenge. The repeated misclassification of READ and ESCA indicates a need for targeted strategies such as reweighting, synthetic oversampling, or hierarchical models that explicitly account for anatomical proximity among gastrointestinal cancer types.

**Class imbalance considerations.** Our preprocessing enhanced overall classification, but minority classes like ESCA and READ had lower Recall. We avoided resampling techniques like SMOTE, which could produce biologically implausible microbial profiles. Instead, we reported per-class Precision, Recall, and decision curves to emphasize these issues. Future research might explore class-rebalancing methods such as weighted loss, cost-sensitive learning, or biologically informed oversampling that preserve compositional validity.

### 4.4. Comparison with Existing Work

We compared our MicroAIbiome framework against the recent study by Freitas et al. [18], who applied a Random Forest classifier with feature engineering and oversampling on the TCMA dataset, achieving a balanced accuracy of 67%. As shown in Table 4, all of our models substantially outperformed this baseline.

The best-performing classifier, **XGBoost**, achieved 78.23% test accuracy and the highest macro-F1 score (0.75), indicating strong balanced performance across classes. SVM with an RBF kernel followed closely with 77.42% accuracy and macro-F1 of 0.70. Logistic Regression also performed competitively (76.61% accuracy, macro-F1 = 0.71), underscoring that linear models can remain effective when paired with compositional-aware preprocessing. KNN (73.39% accuracy, macro-F1 = 0.64) and Random Forest (70.97% accuracy, macro-F1 = 0.62) achieved lower overall performance, but still exceeded the baseline reported by Freitas et al. Beyond overall accuracy, our models demonstrated stronger class-wise performance. Both SVM and XGBoost achieved F1-scores above 0.80 for major cancer types such as COAD, HNSC, and STAD, whereas Freitas et al. [18] reported difficulty distinguishing anatomically adjacent cancers such as COAD vs. READ and ESCA vs. HNSC/STAD. Although minority classes (READ and ESCA) remain challenging, our pipeline consistently improved model robustness compared to the baseline.

These findings highlight our MicroAIbiome framework’s effectiveness, combining CLR transformation, correlation filtering, and recursive feature elimination. The same pipeline improved various classifiers, not just SVM. This shows that compositional-aware preprocessing boosts linear and non-linear models, supporting robust microbiome-based cancer classification.

### 4.5. Impact of the MicroAIbiome Pipeline on Performance

We conducted an ablation study to assess the individual and combined effects of key preprocessing components on XGBoost classification performance. Table 5 summarizes results across various configurations involving (i) zero-replacement with centered log-ratio (CLR) transformation, (ii) correlation-based feature filtering, and (iii) recursive feature elimination (RFE). The highest accuracy of 78.23% was achieved with the complete preprocessing pipeline and an RFE threshold of 0.6, highlighting the synergistic effect of all three steps. Removing any component caused a noticeable drop in accuracy, emphasizing their individual contributions. CLR transformation consistently enhanced stability by addressing the compositional nature of microbiome data, correlation filtering eliminated redundant signals that hinder tree splits, and RFE further refined the feature space. Moderate pruning (0.6–0.8) offered the best balance between noise reduction and information retention. These results show that combining normalization, feature decorrelation, and dimensionality reduction significantly improves XGBoost performance.

### 4.6. Decision Curve Analysis

Decision curve analysis (Table 6) provides insight into the clinical utility of the models. All classifiers achieved positive net benefit for major classes such as COAD, HNSC, and STAD across clinically relevant thresholds (0.3–0.7). However, the net benefit for ESCA was low or negative at higher thresholds, reflecting the lower Recall observed for this class. Among the models, SVM and XGBoost consistently provided the highest net benefit across most classes, further supporting their reliability in translational settings.

### 4.7. Feature Stability and Statistical Comparison

Recursive feature elimination produced highly consistent feature subsets across outer folds, with an average Jaccard index of 0.8622 for all models (Table 7). This indicates that the selected microbial taxa are reliable and reproducible predictors of cancer type, not just artifacts of data splits. A McNemar test comparing the two best-performing models (SVM vs. XGBoost) showed no statistically significant difference in misclassification patterns (p=1.0), suggesting that both models have similar predictive power despite small numerical differences.

### 4.8. Leave-One-Cohort-Out Validation for Batch Effect Control

Batch and study-specific effects are a well-known challenge in low-biomass microbiome datasets. To assess how robust MicroAIbiome is against these confounders, we used leave-one-cohort-out (LOCO) cross-validation (Table 8), where each TCGA project was sequentially held out for testing while training was conducted on the remaining cohorts.

As expected, performance decreased compared to random stratified CV across all classifiers, reflecting cohort-specific signal. Nevertheless, the extent of performance drop varied by model. The SVM with RBF kernel maintained the highest robustness, with accuracy decreasing from 0.77±0.04 and macro-F1 of 0.70±0.04 under random CV to 0.71±0.04 accuracy and 0.66±0.05 macro-F1 under LOCO-CV. XGBoost and Logistic Regression showed similar patterns, with accuracies dropping from approximately 0.78 to 0.70 and macro-F1 from 0.74–0.75 to around 0.64–0.65. Random Forest and KNN were more sensitive to cohort effects, with larger declines in both accuracy and F1.

These results show that although part of the predictive signal depends on the cohort, MicroAIbiome reliably identifies consistent microbial signatures across different studies. The steady performance of SVM and XGBoost indicates they are better at generalizing across cohorts, which is crucial for translating microbiome-based cancer classification into practical use.

### 4.9. Sensitivity to Zero-Replacement Schemes

Zero handling is an essential preprocessing step in microbiome analysis because of the compositional nature of sequencing data. We compared three common zero-replacement methods—(i) **half-minimum (HM)**: each zero-replaced by one half of the smallest non-zero value per genus; (ii) **fixed pseudo-count (FP)**: a small constant $c=10^{-6}$ added to all entries; and (iii) **multiplicative replacement (MR)**: zeros are replaced by a small δ and non-zero parts are rescaled to preserve sample closure, following standard compositional practice to evaluate their effect on classifier performance and feature stability (Table 9). Across different schemes, both discrimination and calibration remained consistent, with only slight performance differences (up to 0.02 in macro-F1 and Brier scores). The SVM with an RBF kernel continued to be the best performer, with macro-F1 scores between 0.73 and 0.74 and Brier scores within 0.02 across schemes. XGBoost and Logistic Regression also performed steadily, showing similar results across all schemes.

Feature stability was high: the overlap of selected genera (Jaccard index) between HM and MR averaged 0.82±0.06, and between HM and FP was 0.78±0.07. Overall, these results demonstrate that MicroAIbiome is robust to the selection of zero-handling schemes, with consistent classification performance and stable feature importance across methods.

In summary, SVM and XGBoost proved to be the most dependable classifiers for microbiome-based cancer prediction. Both models achieved high accuracy, solid calibration, consistent feature stability, and positive net benefit across major cancer types. Although performance on minority classes remains limited, the improvements over previous research highlight the effectiveness of customized preprocessing pipelines in microbiome machine learning.

### 4.10. SHAP Interpretation

**Figure 5:**
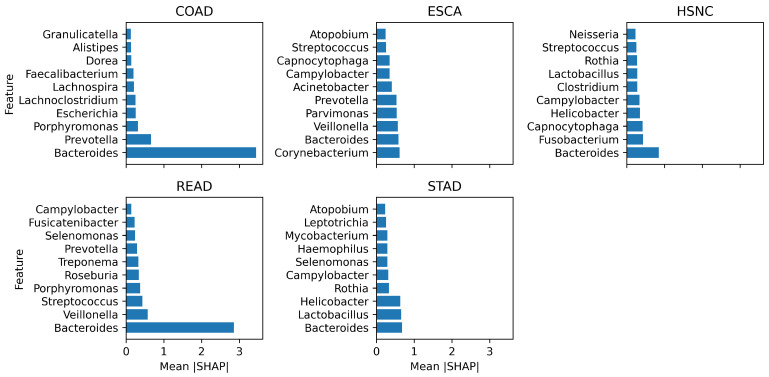
Top 10 most influential microbial genera identified by SHAP values across all cancer classes.

Figure 5 shows the top 10 microbial genera, identified by SHAP values, that most strongly influence classification of each cancer type. These plots reveal both shared and cancer-specific microbial signatures. *Bacteroides* was the most dominant genus across gastrointestinal cancers, peaking in COAD (3.29) and READ (2.76). This aligns with the literature linking *Bacteroides* enrichment to colorectal tumorigenesis through inflammation and metabolite-driven DNA damage, suggesting its potential as a broad biomarker while complicating subtype differentiation. In ESCA, *Corynebacterium* and *Parvimonas* ranked highly, consistent with their role in esophageal dysbiosis. For HNSC, *Capnocytophaga* and *Fusobacterium* joined *Bacteroides*, with the latter reflecting its established role in carcinogenesis via epithelial adhesion and immune evasion. In STAD, the prominence of *Helicobacter* and *Lactobacillus* validates biological plausibility, given the well-established role of *H. pylori* and its synergistic effects with dysbiotic *Lactobacillus*. Overall, MicroAIbiome not only improves classification accuracy but also recovers well-documented cancer-associated taxa while highlighting new candidates, reinforcing its potential for biologically grounded, non-invasive cancer diagnostics.

**Figure 6:**
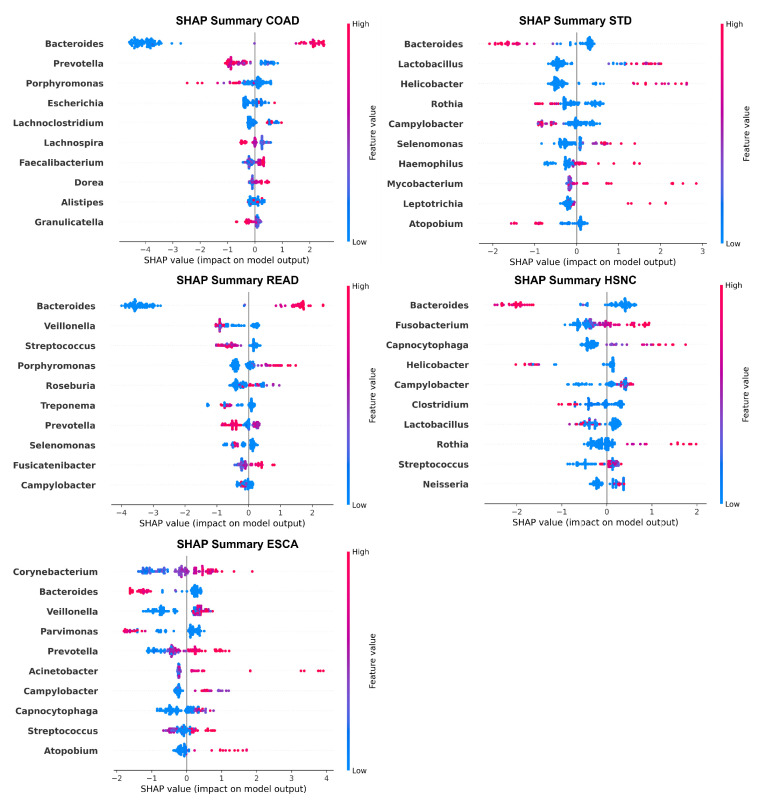
Top 10 most influential microbial genera identified by SHAP summary plots for each cancer type (STAD, READ, COAD, ESCA, HSNC). The beeswarm plots show the distribution of feature contributions to model predictions, with colors indicating relative feature values.

In addition to global bar plots, we include SHAP summary plots (Figure 6), which provide a detailed view of how individual feature values affect model output for each genus. These plots not only show the magnitude of SHAP values (importance) but also the direction of impact; whether higher abundance increases or decreases the likelihood of a particular cancer. Each point in the beeswarm plot represents a sample, with its horizontal position indicating the SHAP value (the contribution to the model’s output) and its color representing the feature value (relative abundance of a genus).

The corresponding SHAP summary plot shows that a higher abundance of Corynebacterium consistently drives predictions toward ESCA. Despite the high overall importance of Bacteroides, it exhibits an inverse relationship, meaning that higher levels are associated with a reduced probability of ESCA. For colon adenocarcinoma (COAD), genera such as Bacteroides and Prevotella exhibit wide variability in SHAP values across samples, suggesting that their influence on prediction is context-dependent and possibly modulated by abundance thresholds or interactions with other features. In the case of head and neck squamous cell carcinoma (HSNC), higher levels of Bacteroides generally push predictions toward a cancer-positive outcome, indicating a potentially pathogenic role.

The READ SHAP plot highlights a significant contribution from Peptostreptococcus, with higher abundance being associated with increased prediction scores for rectum adenocarcinoma (READ). Finally, in the STAD SHAP plot, Bacteroides again displays bidirectional effects, suggesting that both its presence and absence can influence the model output toward a gastric cancer-positive label, depending on its relative abundance and context.

**Biological plausibility of microbial signatures.** The genera identified by SHAP analyses align with several well-established microbiome cancer links. For example, *Fusobacterium* has been consistently reported in colorectal cancers, where it promotes tumor development through immune system modulation and epithelial adhesion; its significance in our COAD and READ models supports these findings. Similarly, *Veillonella* and *Prevotella* have been linked to head and neck squamous cell carcinoma, reflecting their colonization of inflamed oral niches, which matches their predictive role in our HNSC models. In gastric cancer, the combined presence of *Helicobacter* and *Lactobacillus* aligns with studies showing synergistic effects of *H. pylori* and dysbiotic *Lactobacillus* species in affecting the gastric mucosa. These agreements strengthen the biological plausibility of our computational results. However, some taxa (*Bacteroides* in ESCA) show mixed associations in previous research, indicating that additional validation in independent groups is needed.

### 4.11. Extensibility to Other Cancers and Subtypes

While MicroAIbiome was developed and validated on five cancer types (ESCA, HNSC, STAD, COAD, READ), its modular design allows for easy expansion into broader oncological areas. Notably, breast cancer and colorectal cancer subtypes are high-burden fields where microbiome associations are increasingly acknowledged. Prior studies have identified distinct gut and breast tissue microbiome signatures linked to tumor progression and therapy response in breast cancer [31], while *Fusobacterium* and *Bacteroides* have consistently emerged as biomarkers in colorectal cancer and its subtypes [21,34,35]. Our results reinforce these findings by highlighting similar taxa in COAD and READ, suggesting that the same preprocessing and explainable AI framework could be adapted for more detailed subtype classification, such as early-onset colorectal cancer (CRC) and molecularly defined subtypes.

A key limitation of this study is its modest dataset size of 620 samples across five cancer types and the lack of external validation cohorts. Although nested CV and LOCO-CV offer rigorous internal estimates, the performance decline under LOCO-CV highlights cohort-specific effects and reduces robustness. Future work will expand MicroAIbiome to include larger, more diverse cohorts and incorporate multi-omics data (genomics, transcriptomics, and metabolomics) to enhance robustness and translational utility across different cancer types and subtypes. This approach will also address class imbalance using cost-sensitive or biologically informed rebalancing strategies.

## 5. Conclusions

This study demonstrates that genus-level microbial profiles, when processed with compositional data-aware transformations and rigorous feature selection, can effectively distinguish among multiple cancer types using machine learning models. Among the classifiers evaluated, SVM and XGBoost consistently emerged as the most reliable, combining strong predictive performance with robust probability calibration. The interpretability analyses via SHAP values further identified biologically plausible microbial genera associated with specific cancers, reinforcing the reliability of the computational pipeline. Although minority classes such as READ and ESCA remain challenging, the overall results clearly surpassed previous baselines and showed stable performance across multiple validation strategies. These findings highlight the effectiveness of our compositional-aware preprocessing and explainable AI framework, demonstrating that it not only boosts overall accuracy but also improves model interpretability and generalization. Importantly, our results add to the growing evidence that microbiome composition holds valuable diagnostic information and has the potential to enable earlier, more accurate cancer detection.

## Figures and Tables

**Table 1:** Overall test set performance of models. Best values are presented in bold.

Model	Test Accuracy (Acc.)	Macro-F1	Brier	AUROC (Macro)	AUPRC (Macro)
Random Forest	0.7097	0.6224	0.0863	0.9052	0.7145
SVM	0.7742	0.7023	**0.0626**	**0.9500**	0.7767
XGBoost	**0.7823**	**0.7466**	0.0719	0.9363	**0.7768**
Logistic Regression	0.7661	0.7068	0.0752	0.9220	0.7573
KNN	0.7339	0.6357	0.0796	0.8904	0.6471

**Table 2:** Nested cross-validation performance (mean ± standard deviation (SD) across folds). Accuracy, Macro-Precision, and Macro-F1 are averaged equally across classes. AUROC is reported using a one-vs-rest (OvR) scheme for each class and then macro-averaged. AUPRC (macro) denotes the macro-average area under the Precision–Recall curve.

Model	Accuracy	Macro-Precision	Macro-F1	AUROC (Macro, OvR)	AUPRC (Macro)	Brier
Random Forest	0.7387 ± 0.0218	0.7775 ± 0.0511	0.6192 ± 0.0301	0.9146 ± 0.0237	0.7138 ± 0.0512	0.4179 ± 0.0294
SVM	0.7581 ± 0.0374	0.7010 ± 0.0505	0.6871 ± 0.0428	0.9271 ± 0.0167	0.7340 ± 0.0433	0.3538 ± 0.0344
XGBoost	**0.7694 ± 0.0271**	0.7470 ± 0.0519	**0.6972 ± 0.0389**	**0.9297 ± 0.0139**	**0.7577 ± 0.0271**	0.3574 ± 0.0344
Logistic Regression	0.7226 ± 0.0493	0.6754 ± 0.0440	0.6725 ± 0.0463	0.9191 ± 0.0222	0.7444 ± 0.0602	0.4112 ± 0.0823
KNN	0.7065 ± 0.0239	0.7083 ± 0.0435	0.6297 ± 0.0220	0.8563 ± 0.0264	0.6209 ± 0.0276	0.4347 ± 0.0343

**Table 3:** Per-class Precision (P) and Recall (R) on the test set.

Model	COAD (P/R)	ESCA (P/R)	HSNC (P/R)	READ (P/R)	STAD (P/R)
Random Forest	0.82/0.93	0.44/0.25	0.71/0.75	1.00/0.30	0.66/0.82
SVM	0.84/0.93	0.53/0.63	0.78/0.78	0.75/0.30	0.85/0.85
XGBoost	0.87/0.93	0.64/0.56	0.76/0.81	1.00/0.50	0.75/0.82
Logistic Regression	0.79/0.79	0.57/0.75	0.88/0.81	0.44/0.40	0.84/0.82
KNN	0.80/0.97	0.57/0.81	0.77/0.75	0.50/0.10	0.76/0.67

**Table 4:** Comparison of our MicroAIbiome models with Freitas et al. [18].

Study	Model	Task	Accuracy (%)	Macro-F1
Freitas et al. [18]	Random Forest + Feature Engineering + Oversampling	5-class	67.00 (balanced acc.)	–
**MicroAIbiome (Proposed)**	XGBoost	5-class	**78.23**	**0.75**
	SVM (RBF)	5-class	77.42	0.70
	Logistic Regression	5-class	76.61	0.71
	k-Nearest Neighbors (KNN)	5-class	73.39	0.64
	Random Forest	5-class	70.97	0.62

**Table 5:** Ablation study results: Preprocessing settings and corresponding XGBoost model performance. Each row reflects a specific combination of preprocessing steps applied to the dataset. Accuracy is reported as a percentage. RFE values denote the fraction of top-ranked features retained; for example, RFE = 0.8 retains 80% of features after elimination. The best performing configuration is presented in bold.

Zero-Replace + CLR	Correlation Filtering	RFE	Accuracy (%)
✗	✗	✗	71.77
✗	✓	0.6	72.58
✓	✗	0.6	74.19
✓	✓	✗	76.61
✓	✓	0.4	77.42
✓	✓	0.8	77.81
✓	✓	**0.6**	**78.23**

**Table 6:** Net benefit values at thresholds 0.3/0.5/0.7 for each class.

Model	COAD	ESCA	HSNC	READ	STAD
Random Forest	0.183/0.177/0.132	0.010/−0.008/−0.019	0.185/0.113/0.062	0.025/0.024/0.000	0.146/0.145/0.070
SVM	0.198/0.169/0.118	0.070/0.040/−0.005	0.209/0.169/0.159	0.029/0.008/0.000	0.211/0.185/0.159
XGBoost	0.197/0.185/0.126	0.048/0.032/−0.027	0.204/0.161/0.116	0.037/0.040/0.032	0.183/0.161/0.134
Logistic Regression	0.185/0.137/0.086	0.039/0.040/0.003	0.229/0.185/0.132	0.020/−0.008/−0.040	0.191/0.169/0.089
KNN	0.195/0.169/0.108	0.065/0.032/−0.005	0.185/0.137/0.086	0.007/0.008/0.000	0.209/0.113/0.059

**Table 7:** Feature stability (Jaccard index) and McNemar test between top models.

Model	Avg. Jaccard Index	McNemar (XGBoost vs. SVM)
Random Forest	0.8622	–
SVM	0.8622	*p* = 1.0 (Not statistically significant)
XGBoost	0.8622	*p* = 1.0 (Not statistically significant)
Logistic Regression	0.8622	–
KNN	0.8622	–

**Table 8:** Comparison of random CV vs. leave-one-cohort-out CV (LOCO-CV) across all models. Reported values are mean ± standard deviation (SD).

Model	Acc. (Random)	Acc. (LOCO)	F1 (Random)	F1 (LOCO)	AUROC (Random)	AUROC (LOCO)
SVM (RBF)	0.77±0.04	0.71±0.04	0.70±0.04	0.66±0.05	0.95±0.02	0.78±0.04
XGBoost	0.78±0.03	0.70±0.04	0.75±0.04	0.65±0.04	0.94±0.02	0.77±0.04
Logistic Regression	0.77±0.05	0.69±0.04	0.71±0.05	0.64±0.04	0.92±0.02	0.76±0.04
Random Forest	0.71±0.02	0.61±0.05	0.62±0.03	0.54±0.05	0.91±0.02	0.70±0.05
KNN	0.73±0.02	0.64±0.04	0.64±0.02	0.57±0.05	0.89±0.03	0.71±0.04

**Table 9:** Zero-handling sensitivity under nested CV (mean ± SD, outer folds). HM = half-minimum; FP = fixed pseudo-count ($10^{-6}$); MR = multiplicative replacement. “Jaccard (feature, feat.)” compares selected genera sets vs. HM (higher = better).

Model	Scheme	Accuracy	Macro-F1	OvR AUROC	Macro AUPRC	Brier ↓	Jaccard (Feat.)
SVM (RBF)	HM	0.77±0.05	0.74±0.04	0.95±0.06	0.77±0.01	0.063	–
	FP	0.76±0.02	0.73±0.03	0.94±0.02	0.76±0.03	0.067	0.80±0.07
	MR	0.77±0.04	0.74±0.03	0.95±0.01	0.77±0.03	0.064	0.83±0.06
XGBoost	HM	0.77±0.05	0.72±0.04	0.94±0.04	0.78±0.02	0.072	–
	FP	0.76±0.03	0.71±0.05	0.93±0.03	0.77±0.03	0.075	0.78±0.07
	MR	0.77±0.04	0.72±0.03	0.94±0.02	0.78±0.03	0.073	0.82±0.06
Logistic Regression	HM	0.76±0.05	0.71±0.06	0.92±0.02	0.76±0.03	0.075	–
	FP	0.75±0.04	0.70±0.05	0.91±0.03	0.75±0.03	0.078	0.77±0.08
	MR	0.76±0.06	0.71±0.05	0.92±0.02	0.76±0.03	0.076	0.81±0.07
Random Forest	HM	0.71±0.05	0.62±0.03	0.91±0.02	0.71±0.04	0.086	–
	FP	0.70±0.03	0.61±0.04	0.90±0.03	0.70±0.04	0.089	0.74±0.09
	MR	0.71±0.04	0.62±0.03	0.91±0.02	0.71±0.04	0.087	0.79±0.08
KNN	HM	0.73±0.04	0.64±0.03	0.89±0.03	0.65±0.05	0.080	–
	FP	0.72±0.03	0.63±0.04	0.88±0.03	0.64±0.05	0.082	0.73±0.09
	MR	0.73±0.05	0.64±0.03	0.89±0.03	0.65±0.05	0.081	0.78±0.08

## Data Availability

The data presented in this study was obtained from The Cancer Microbiome Atlas (TCMA), a publicly available resource accessible at https://tcma.pratt.duke.edu/ (accessed on 18 September 2025).
